# Glucagon-like peptide-1 mimetics, optimal for Asian type 2 diabetes patients with and without overweight/obesity: meta-analysis of randomized controlled trials

**DOI:** 10.1038/s41598-017-16018-9

**Published:** 2017-11-22

**Authors:** Fang Zhang, Lizhi Tang, Yuwei Zhang, Qingguo Lü, Nanwei Tong

**Affiliations:** Division of Endocrinology and Metabolism, West China Hospital, Sichuan University, Chengdu, 610041 China

## Abstract

Glucagon-like peptide-1 receptor agonists (GLP-1RAs) are desirable for diabetes, especially in patients with overweight/obesity. We aimed to determine whether GLP-1RAs exhibit different glucose-lowering efficacies between Asian type 2 diabetes (T2D) patients with and without overweight/obesity. Randomized controlled trials were searched in EMBASE, MEDLINE, CENTRAL, and ClinicalTrials.gov. Studies published in English with treatment duration ≥12 weeks and information on HbA1c changes were included. The studies were divided into normal body mass index (BMI) and overweight/obese groups according to baseline BMI. Among 3190 searched studies, 20 trials were included in the meta-analysis. The standardized mean differences in HbA1c change, fasting glucose change, and postprandial glucose change were equivalent between normal BMI and overweight/obese studies (p > 0.05). The relative risk of HbA1c < 6.5% target achievement in normal BMI trials (7.93; 95% confidence interval: 3.27, 19.20) was superior to that in overweight/obesity trials (2.23; 1.67, 2.97), with a significant difference (p = 0.020). Body weight loss (p = 0.572) and hypoglycemic risk(p = 0.920) were similar in the two groups. The glucose-lowering effects of GLP-1RAs were equivalent among Asian T2D patients. With their advantages for weight-loss or weight-maintenance, GLP-1RAs are optimal medicines for Asian T2D patients with and without overweight/obesity.

## Introduction

Diabetes and its complications are increasing epidemics in Asia, and pose major challenges to health-care systems and economics. It is estimated that Asia will become the region with the highest population of diabetes patients worldwide by 2025, wherein the numbers of diabetes patients in India, China, and Japan will reach 69.6, 59.3, and 7.2 million, respectively^1,2^. Overweight and obesity are involved in the etiology of type 2 diabetes (T2D) in Asia^3^. Approximately 3.32 million T2D incidences in Chinese adults were attributable to overweight/obesity in 2010^4^. Thus, anti-diabetic drugs — with not only hypoglycemic efficacy, but also influence on body weight — are pivotal for T2D patients.

Glucagon-like peptide-1 receptor agonists (GLP-1RAs), which have valid glucose-lowering efficacies as well as notable weight-loss effects, are beneficial for diabetes therapy, especially for patients with overweight/obesity. A recent meta-analysis implied that GLP-1RAs lower HbA1c more effectively in Asians than in non-Asians^5^. Moreover, the HbA1c-lowering efficacies of GLP-1RAs were greater in studies with average body mass index (BMI) < 30 kg/m^2^ than those in studies with average BMI ≥ 30 kg/m^2^. It is considered that the difference in GLP-1RA hypoglycemic effects between Asian and non-Asian studies can be largely ascribed to their different baseline BMIs. Another meta-analysis suggested that lower BMI may be a predictor of good response to dipeptidyl peptidase-4 (DPP-4) inhibitors^6^. Similar to the case for GLP-1RAs, the meta-analysis demonstrated that DPP-4 inhibitors decrease HbA1c more effectively in Asians than in non-Asians. DPP-4 inhibitors have a similar hypoglycemic mechanism to GLP-1RAs, and increase the level of intact GLP-1. Taken together, these findings revealed that compared with non-Asian T2D patients, GLP-1 mimetics are more efficacious in Asian T2D patients. However, within Asian individuals, it remains unknown whether different BMIs lead to different HbA1c-lowering efficacies of GLP-1RAs. Therefore, using previous randomized controlled trials (RCTs), we performed a systematic review and meta-analysis to examine the differences in GLP-1RA glucose-lowering effects and safety between normal BMI and overweight/obese BMI Asian T2D patients.

## Methods

The primary aim of our systematic review was to assess the difference in GLP-1RA HbA1c-lowering effects in Asian T2D patients with and without overweight/obesity. Besides HbA1c, the changes in fasting plasma glucose (FPG), 2-hour postprandial glucose (2hPG), and body weight were considered. A safety comparison between the two BMI populations was also carried out. Our study was conducted in accordance with the Preferred Reporting Items for Systematic Reviews and Meta-Analyses (PRISMA)^7^.

### Search Strategy, Overweight/obesity Criteria and Study Selection

We searched EMBASE, MEDLINE, Cochrane Central Register of Controlled Trials (CENTRAL), and ClinicalTrials.gov (http://www.clinicaltrials.gov) for relevant studies according to the following search-term strategy: T2D AND (GLP-1RA OR GLP-1 derivative OR GLP-1 analogue OR exenatide OR liraglutide OR lixisenatide OR dulaglutide OR taspoglutide OR semaglutide OR albiglutide) AND RCT. The last search was performed on 04 April, 2017.

Overweight and obesity were defined as BMI ≥ 25 kg/m^2^ and ≥30 kg/m^2^ in Japanese trials, and BMI ≥ 24 kg/m^2^ and ≥28 kg/m^2^ in Chinese trials, respectively^8,9^. For studies containing more than one Asian race/country, overweight and obesity were defined as BMI ≥ 23 kg/m^2^ and ≥27.5 kg/m^2^, respectively^9^. Based on the baseline BMI values in the GLP-1RA intervention groups, we categorized the eligible studies into normal BMI and overweight/obese BMI studies.

The initially screened articles were only considered for inclusion in our systematic review when they met all of the following criteria: (1) the RCTs were published in English; (2) all participants were Asian adults diagnosed with T2D; (3) GLP-1RAs as monotherapy or add-on drug were compared with other anti-diabetic drugs or placebo; (4) intervention duration was at least 12 weeks; (5) baseline BMI information in each intervention group was described; and (6) mean and standard deviation (SD) of changes in glucose profiles could be derived. In addition, studies with total sample size <50 patients were excluded from the final meta-analysis. Duplications or extensions originating from the same trial were also excluded. Two authors (F. Z. and Y. Z.) carried out the literature searches and study selections independently and any divergence in opinions was resolved by discussion.

### Data Extraction

Two authors (F. Z. and L. T.) independently extracted data, evaluated study quality, and assessed bias risk in the eligible articles. Any discrepancy was resolved by mutual checks or consensus of all authors. First author name, publication year, racial heritages of patients, study sample size, mean GLP-1RA treatment duration, mean diabetes duration, mean age, sex constitution, mean BMI at baseline, protocols for GLP-1RA and control therapies, changes in HbA1c, FPG, 2hPG, and body weight, numbers of participants who achieved HbA1c < 7.0% or <6.5% target goals, and numbers of patients who experienced adverse events were collected. The following equations were available to calculate missing data, when the relevant changes in outcome measurements were not presented in the literature:

1 $${\bar{X}}_{change}=\,{\bar{X}}_{post-treatment}-\,{\bar{X}}_{baseline}$$

2 $$S{D}_{change}=\,\sqrt{{(S{D}_{baseline})}^{2}+{(S{D}_{post-treatment})}^{2}-2\times r\times S{D}_{baseline}\times S{D}_{post-treatment}}$$

where *r* is a correlation coefficient with a value of 0.4^10^. If only the standard error (SE) or 95% confidence interval (CI) was displayed, we obtained SD _Change_ by the methods recommended in the *Cochrane Handbook for Systematic Reviews of Interventions* (version 5.1)^11^. When a trial contained subgroups with diverse doses of GLP-1RA or several controlled agents, we divided the trial into respective comparison pairs: each pair included one dose of GLP-1RA versus one controlled drug.

### Risk of Bias Assessment

A funnel plot and Egger’s test were used to assess publication bias in all studies involved in the meta-analysis. The quality of each study in the systematic review was evaluated by the Cochrane Collaboration tool, which is based on bias risks from seven aspects: sequence generation randomness, allocation concealment, blinding of participants and staff, blinding outcome assessment, outcome data incompleteness, selective reporting and other bias^11^. Low risk, high risk, and unclear risk were the three ranks in each category.

### Statistical Analysis

The differences in glucose-lowering efficacies and safety outcomes between the normal BMI and overweight/obese BMI groups were detected. All data were analyzed using Stata version 13.0 (Stata Corp. College Station, TX, USA). Continuous outcomes were expressed as standardized mean difference (SMD) and dichotomous data were represented as relative risk (RR) in the meta-analysis, both with a 95% CI. The chi-square test and I^2^ statistics were used to evaluate intertrial heterogeneity. If heterogeneity was detected (p ≤ 0.1; I^2^ > 50%), a random-effects model was adopted. Otherwise, if heterogeneity was not detected (p > 0.1; I^2^ ≤ 50%), a fixed-model was adopted. When statistical heterogeneity occurred, we initially analyzed its source, and then performed a sensitivity analysis to examine the robustness of the results. For the primary efficacy outcome, HbA1c change, if a heterogeneous result was robust, a meta-regression analysis was carried out to further investigate which prespecified clinical characteristics may be potential sources of the intertrial heterogeneity. We evaluated the changes in FPG, 2hPG, and body weight in the two groups as well as the numbers of HbA1c target goal achievements and numbers of adverse events. Comparisons of SMDs or RRs between the two groups were performed by meta-regression. Values of p < 0.05 were considered statistically significant unless otherwise stated. Subgroup analyses were conducted in studies with liraglutide intervention or trials involving comparisons of GLP-1RAs versus placebo.

## Results

### Literature Selection and Study Characteristics

**Figure 1:**
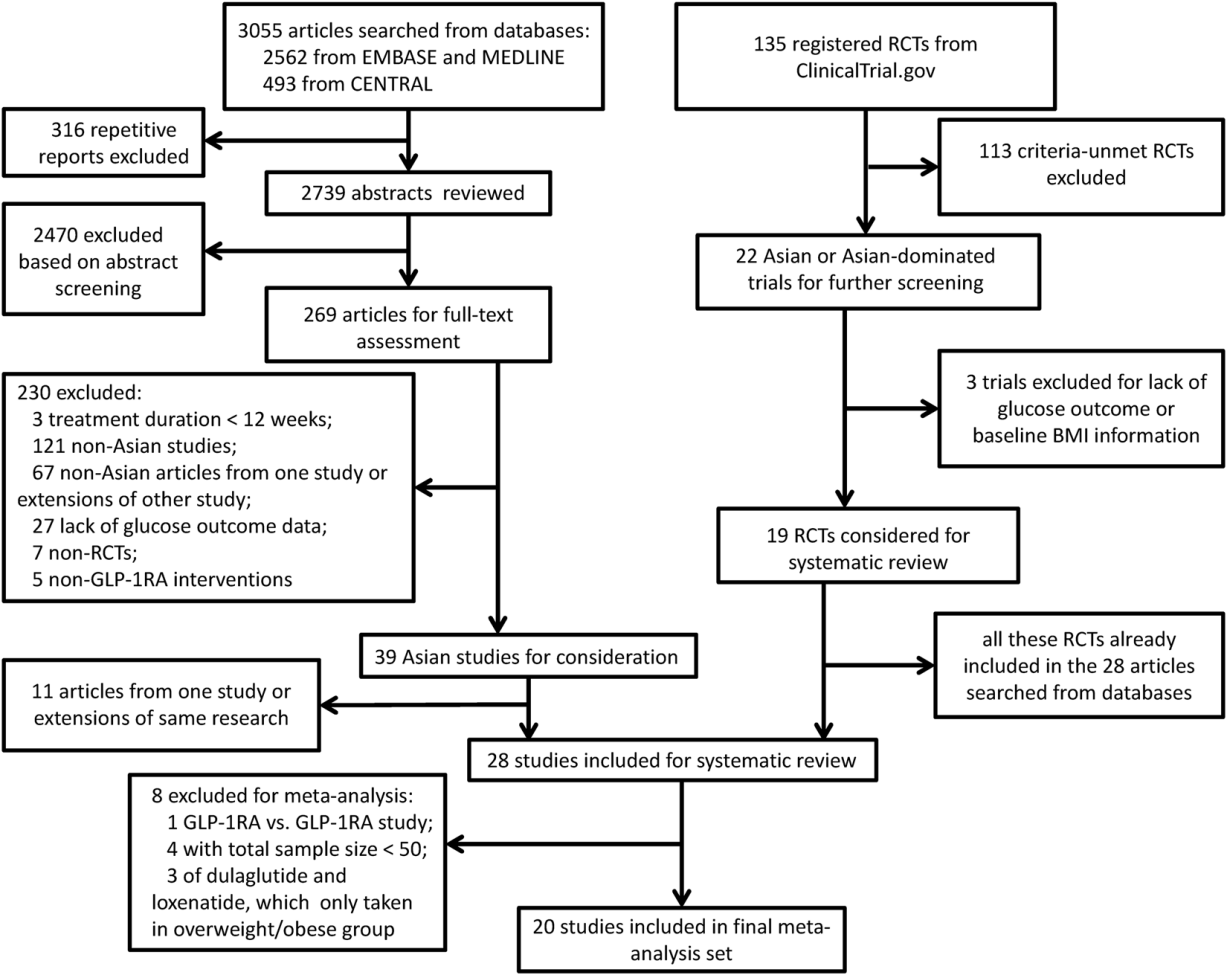
Flowchart of trial selection in the systematic review and meta-analysis. RCT, randomized controlled trial; GLP-1RA, glucagon-like peptide-1 receptor agonist; BMI, body mass index.

Among the 3055 articles obtained in the primary searches from MEDLINE, EMBASE, and CENTRAL databases and the 135 potentially relevant trials identified in ClinicalTrials.gov, a total of 28 published studies that met the inclusion criteria were included in the systematic review. The study selection process is shown in Fig. 1. One of the 28 trials was excluded from the meta-analysis, because of its design for GLP-1RA versus GLP-1RA comparison^12^. Four studies with sample size <50 patients^13–16^ and three studies with GLP-1RA dulaglutide or loxenatide that only included overweight/obese BMI patients (without the comparator studies which included normal BMI patients)^17–19^ were also excluded. Thus, 20 studies with exenatide, liraglutide and lixisenatide were used for the final meta-analysis, among which four studies were categorized into the normal BMI group^20–23^, fourteen studies were categorized into the overweight/obese BMI group^24–37^, and two trials with subgroups of diverse BMIs were included in both groups^38,39^. The meta-analysis involved 3281 individuals in the GLP-1RA treatment group and 2510 patients in the control group. The characteristics of the included studies are summarized in Table 1.

**Table 1:** Characteristics of the included studies (n = 28).

Study	Sample size	Racial heritage of participants	Treatment duration (weeks)	Diabetes duration (years (SD))	Mean age (years (SD))	Female (%)	Baseline BMI (kg.m-2 (SD))		Intervention	Control
	I/C			I/C	I/C	I/C	I	C		
GLP-1RA versus other anti-diabetic drugs studies										
*Overweight*/*obese BMI studies*										
Araki *et al*. 2015^17^	181/180	Japan	26	8.9 (6.7)/8.8 (6.1)	57.5 (10.5)/56.1 (11.3)	31/26	26.1 (3.6)	25.9 (3.9)	Dulaglutide 0.75 mg QW + sulphonylureas ± biguanides	Insulin glargine + sulphonylureas ± biguanides
Xu *et al*. 2014^24^	Exenatide: 142/Insulin: 138; Pioglitazone: 136	China	48	newly diagnosed	Exenatide: 49.89 (9.64)/Insulin: 51.39 (9.65); Pioglitazone: 49.66 (8.86)	Exenatide: 32.7/Insulin: 38.6; Pioglitazone: 44.9	25.9 (0.3)	Insulin: 25.4 (0.3); Pioglitazone: 25.9 (0.3)	Exenatide 5 ug BID (4 weeks) and 10 ug BID (44 weeks)	Insulin Lispro 25 R BID; or Pioglitazone 30 mg QD (4 weeks) and 45 mg QD (44 weeks)
Chen *et al*. 2017^18^	PEX 168 100ug: 41; PEX 168 200 ug: 39/Placebo: 38	China	12	PEX 168 100 ug: 4.4 (6.4); PEX 168 200 ug: 4 (5.7)/Placebo: 6.5 (7.9)	PEX 168 100 ug: 52.6 (8.4); PEX 168 200 ug: 49.8 (10.9)/Placebo: 53.5 (10.2)	PEX 168 100 ug: 46.34; PEX 168 200 ug: 43.59/Placebo: 31.58	PEX 168 100 ug: 27.2 (3.6); PEX 168 200 ug: 26.3 (3.3)	27.2 (4.5)	PEX 168 100 ug QW or 200 ug QW + Glucophage 1500 mg/d	Placebo + Glucophage 1500 mg/d
Gao *et al*. 2009^25^	234/232	China (Mainland and Taiwan), India, Korea	16	8 (6)/8 /(5)	55 (9)/54 (9)	52/59	26.4 (3.2)	26.1 (3.4)	Exenatide 5 ug BID (4 weeks) and 10 ug BID (12 weeks) + metformin ± sulphonylureas	Placebo + metformin ± sulphonylureas
Inagaki *et al*. 2012^26^	215/212	Japan	26	8.86 (6.06)/9.21 (5.99)	57.07 (10.44)/56.44 (11.16)	34/30.2	26.11 (4.03)	28.18 (3.77)	Exenatide 2 mg QW + biguanide ± thiazolidinederevative	Insulin glargine + biguanide ± thiazolidinederevative
Kadowaki *et al*. 2011^27^	Exenatide 5 ug: 72; Exenatide 10 ug: 72/Placebo: 35	Japan	24	Exenatide 5 ug: 12.2 (6.3); Exenatide 10 ug: 11.6 (7.0)/Placebo: 12.4 (6.5)	Exenatide 5 ug: 58.5 (9.3); Exenatide 10 ug: 59.4 (9.8)/Placebo: 56.3 (11.4)	Exenatide 5 ug: 31.9; Exenatide 10 ug: 31.9/Placebo: 31.4	Exenatide 5 ug: 25.0 (4.1); Exenatide 10 ug: 25.8 (3.9)	25.8 (4.2)	Exenatide 5 ug BID or Exenatide 5 ug BID (4 weeks) and 10 ug BID (20 weeks) + sulphonylureas ± other OAD	Placebo + sulphonylureas ± other OAD
Ke *et al*. 2015^13^	20/19	China	12	newly diagnosed	42.3 (9.9)/42.1 (7.6)	26.7/33.3	25.4 (2.8)	25.5 (2.4)	Liraglutide 0.6 mg QD (during hospitalization) and 1.2 mg QD (from discharged from hospital to the end of the 12th week) + short-term CSII	short-termCSII
Li *et al*. 2012^28^	42/42	China	12	9.1 (3.6)/8.9 (3.6)	51.2 (10.5)/52.7 (10.8)	38.1/42.9	30.4 (3.2)	30.3 (3.0)	Liraglutide 0.6 mg QD (1 week) and 1.2 mg QD (11 weeks) + Insulin ± OAD	Insulin ± OAD
Li *et al*. 2014^29^	Liraglutide: 61; Saxagliptin: 60; Vildagliptin: 57	China	24	Liraglutide: 5.8 (2.8)/Saxagliptin: 5.4 (2.7); Vildagliptin: 5.4 (2.2)	Liraglutide: 47.9 (10.8)/Saxagliptin: 47.0 (11.3); Vildagliptin: 46.4 (9.8)	Liraglutide: 41.0/Saxagliptin: 35.0; Vildagliptin: 40.4	26.7 (2.4)	Saxagliptin: 26.3 (2.2); Vildagliptin: 25.9 (1.8)	Liraglutide 0.6 mg QD (1 week) and 1.2 mg QD (23 weeks) ± OAD	Saxagliptin 5 mg QD ± OAD; or Vildagliptin 50 mg BID ± OAD
Miyagawa *et al*. 2015^30^	Dulaglutide: 280; Liraglutide: 137; Placebo: 70	Japan	26	Dulaglutide: 6.8 (5.6)/Liraglutide: 6.3 (6.0); Placebo: 6.3 (5.1)	Dulaglutide: 57.2 (9.6)/Liraglutide: 57.9 (10.4); Placebo: 57.7 (8.3)	Dulaglutide: 19/Liraglutide: 18; Placebo: 21	25.6 (3.6)	Liraglutide: 25.5 (3.5); Placebo: 25.2 (3.2)	Dulaglutide 0.75 mg QW	Liraglutide 0.3 mg QD (1 week), 0.6 mg QD (1 week) and 0.9 mg QD (24 weeks); or Placebo
Seino *et al*. 2012^31^	154/157	Japan, Republic of Korea, Philippines, China (Taiwan)	24	13.7 (7.7)/14.1 (7.7)	58.7 (10.2)/58.0 (10.1)	55.2/49.0	25.4 (3.7)	25.2 (3.9)	Lixisenatide 10 ug QD (1 week), 15 ug QD (1 week) and 20 ug QD (22 weeks) + Insulin ± sulphonylureas	Placebo + Insulin ± sulphonylureas
Seino *et al*. 2016^32^	127/130	Japan	36	14.32 (8.89)/14.69 (8.60)	61.3 (11.0)/59.8 (11.3)	45.7/42.3	26.2 (4.9)	25.2 (4.0)	Liraglutide 0.3 mg QD (1 week), 0.6 mg QD (1 week) and 0.9 mg QD (34 weeks) + Insulin	Placebo + Insulin
Shi *et al*. 2017^14^	18/18	China	12	2.72 (2.77)/1.91 (2.70)	44.4 (11.1)/38.7 (10.3)	26.7/31.3	31.48 (3.09)	31.13 (2.54)	Exenatide 5 ug BID (4 weeks) and 10 ug BID (8 weeks) + metformin 1500–2000 mg/d	Acarbose 50–100 mg TID + metformin 1500–2000 mg/d
Takeshita *et al*. 2015^33^	54/58	Japan	12	NA	64.7 (12.4)	35.2/37.9	25.4 (4.8)	24.5 (4.6)	Liraglutide 0.3 mg QD (3 days), 0.6 mg QD (3 days) and 0.9 mg QD (78 days) ± OAD	Vildagliptin 100 mg/d ± OAD
Tanaka *et al*. 2015^15^	22/24	Japan	24	5.6 (4.2)/4.7 (3.9)	55 (11)/51 (11)	41.0/33.3	28.6 (4.2)	28.7 (3.7)	Liraglutide 0.3 mg QD (1 week), 0.6 mg QD (1 week) and 0.9 mg QD (22 weeks)	Metformin ≥ 1500 mg/d
Terauchi *et al*. 2014^19^	Dulaglutide 0.25 mg: 36; Dulaglutide 0.5 mg: 37; Dulaglutide 0.75 mg: 35/Placebo: 37	Japan	12	Dulaglutide 0.25 mg: 4.3 (3.5); Dulaglutide 0.5 mg: 4.9 (4.0); Dulaglutide 0.75 mg: 4.6 (4.5)/Placebo: 4.7 (4.5)	Dulaglutide 0.25 mg: 52.3 (8.8); Dulaglutide 0.5 mg: 52.5 (9.2); Dulaglutide 0.75 mg: 52.2 (7.8)/Placebo: 51.7 (9.7)	Dulaglutide 0.25 mg: 25.0; Dulaglutide 0.5 mg: 37.8; Dulaglutide 0.75 mg: 20.0/Placebo: 21.6	Dulaglutide 0.25 mg: 26.8 (4.5); Dulaglutide 0.5 mg: 26.7 (3.8); Dulaglutide 0.75 mg: 27.1 (3.7)	27.4 (4.5)	Dulaglutide 0.25 mg QW, or 0.5 mg QW, or 0.75 mg QW	Placebo
Yang *et al*. 2011^34^	Liraglutide 0.6 mg: 231; Liraglutide 1.2 mg: 233; Liraglutdie 1.8 mg: 234/Glimepiride: 231	China, India, South Korea	16	Liraglutide 0.6 mg: 7.4 (5.4); Liraglutide 1.2 mg: 7.5 (5.3); Liraglutdie 1.8 mg: 7.2 (5.2)/Glimepiride: 7.8 (6.1)	Liraglutide 0.6 mg: 53.5 (9.5); Liraglutide 1.2 mg: 53.5 (9.6); Liraglutdie 1.8 mg: 52.7 (9.1)/Glimepiride: 53.6 (9.7)	Liraglutide 0.6 mg: 45.9; Liraglutide 1.2 mg: 45.1; Liraglutdie 1.8 mg: 46.2/Glimepiride: 41.6	Liraglutide 0.6 mg: 25.9 (4.2); Liraglutide 1.2 mg: 25.4 (3.7); Liraglutdie 1.8 mg: 25.8 (3.8)	25.3 (3.7)	Liraglutide 0.6 mg QD or 1.2 mg QD or 1.8 mg QD + metformin 2000 mg/d	Glimepiride 4 mg/d + metformin 2000 mg/d
Yoon *et al*. 2017^16^	10/13	Korea	30	11.1 (5.4)/13.6 (5.8)	48.2 (12.0)/53.8 (9.2)	60.0/53.8	27.9 (3.4)	28.1 (4.4)	Exenatide 5 ug BID (4 weeks) and 10 ug BID (26 weeks) + Insulin glargine + metformin	Insulin lispro TID + Insulin glargine + metformin
Pan *et al*. 2014^35^	196/194	China (Mainland and Hong Kong), Malaysia, Thailand	24	6.5 (4.6)/6.8 (4.8)	54.5 (10.3)/55.1 (10.5)	48.5/53.1	26.8 (3.9)	27.1 (3.8)	Lixisenatide 10 ug QD (2 weeks) and 20 ug QD (22 weeks) + metformin ± sulphonylureas	Placebo + metformin ± sulphonylureas
Yuan *et al*. 2012^36^	33/26	China	26	newly diagnosed	58.5 (10.6)/56.8 (7.6)	48.5/53.9	30.6 (2.8)	29.3 (2.6)	Exenatide 5 ug BID (4 weeks) and 10 ug BID (22 weeks)	Metformin ≥ 1500 mg/d
Zang *et al*. 2016^37^	183/184	China	26	5.3 (4.4)/5.2 (5.4)	51.7 (10.7)/51.4 (11.0)	44.3/36.4	27.3 (3.4)	27.2 (4.0)	Liraglutide 0.6 mg QD (1 week), 1.2 mg QD (1 week) and 1.8 mg QD (24 weeks) + metformin	Sitagliptin 100 mg/d + metformin
*Normal BMI studies*										
Inoue *et al*. 2015^20^	43/45	Japan	24	9.6 (9.5)/8.5 (7.1)	60.1 (12.6)/60.6 (11.5)	46.5/35.6	24.2 (3.7)	24.7 (3.4)	Liraglutide 0.3 mg QD (1 week), 0.6 mg QD (1 week) and 0.9 mg QD (22 weeks)	Insulin detemir + sitagliptin 50 mg/d
Onishi *et al*. 2015^21^	76/51	Japan	24	12.0 (7.8)/12.4 (8.7)	59.3 (10.3)/59.2 (12.1)	36.8/31.4	24.9 (3.2)	26.2 (4.4)	Lixisenatide 10 ug QD (1 week), 15 ug QD (1 week) and 20 ug QD (22 weeks) + sulphonylureas ± metformin	Placebo + sulphonylureas ± metformin
Seino *et al*. 2010^22^	268/132	Japan	24	8.1 (6.7)/8.5 (6.8)	58.2 (10.4)/58.5 (10.4)	32/35	24.5 (3.7)	24.4 (3.8)	Liraglutide 0.3 mg QD (1 week), 0.6 mg QD (1 week) and 0.9 mg QD (22 weeks)	Glibenclamide 1.25 mg/d (4 weeks) and 2.5 mg/d (20 weeks)
Seino *et al*. 2008^23^	Liraglutide 0.1 mg: 45; Liraglutide 0.3 mg: 46; Liraglutide 0.6 mg: 45; Liraglutide 0.9 mg: 44/Placebo: 46	Japan	14	Liraglutide 0.1 mg: 7.15 (5.14); Liraglutide 0.3 mg: 6.78 (4.69); Liraglutide 0.6 mg: 8.87 (6.77); Liraglutide 0.9 mg: 7.62 (4.92)/Placebo: 7.48 (5.65)	Liraglutide 0.1 mg: 56.5 (8.4); Liraglutide 0.3 mg: 56.8 (8.8); Liraglutide 0.6 mg: 60.0 (7.0); Liraglutide 0.9 mg: 55.5 (7.6)/Placebo: 57.5 (8.7)	Liraglutide 0.1 mg: 31.1; Liraglutide 0.3 mg: 30.4; Liraglutide 0.6 mg: 37.8; Liraglutide 0.9 mg: 29.5/Placebo: 40.0	Liraglutide 0.1 mg: 24.26 (2.77); Liraglutide 0.3 mg: 23.93 (3.09); Liraglutide 0.6 mg: 23.74 (2.78); Liraglutide 0.9 mg: 23.59 (3.04)	23.77 (2.63)	Liraglutide 0.1 mg QD, or 0.3 mg QD, or 0.6 mg QD, or 0.9 mg QD	Placebo
*Studies including both overweight/obese and normal BMIs*										
Kadowaki *et al*. 2009^38^	Exenatide 2.5 ug: 38; Exenatide 5 ug: 37; Exenatide 10 ug: 38/Placebo: 40	Japan	12	Exenatide 2.5 ug: 14.8 (10.9); Exenatide 5 ug: 11.3 (6.4); Exenatide 10 ug: 9.6 (6.0)/Placebo: 11.9 (6.0)	Exenatide 2.5 ug: 62.2 (7.8); Exenatide 5 ug: 60.7 (9.8); Exenatide 10 ug: 57.8 (10.4)/Placebo: 60.5 (10.2)	Exenatide 2.5 ug: 29.7; Exenatide 5 ug: 32.4; Exenatide 10 ug: 37.8/Placebo: 25.0	Exenatide 2.5 ug: 24.2 (3.3); Exenatide 5 ug: 25.0 (3.4); Exenatide 10 ug: 26.1 (5.3)	25.8 (4.6)	Exenatide 2.5 ug BID or Exenatide 5 ug BID or Exenatide 5 ug BID (4 weeks) and 10 ug BID (8 weeks) + sulphonylureas ± other OAD	Placebo + sulphonylureas ± other OAD
Kaku *et al*. 2010^39^	Liraglutide 0.6 mg: 88; Liraglutide 0.9 mg: 88/Placebo: 88	Japan	24	Liraglutide 0.6 mg: 9.3 (5.8); Liraglutide 0.9 mg: 11.6 (7.7)/Placebo: 10.1 (7.3)	Liraglutide 0.6 mg: 59.1 (10.3); Liraglutide 0.9 mg: 61.3 (11.0)/Placebo: 10.1 (7.3)	Liraglutide 0.6 mg: 40; Liraglutide 0.9 mg: 33/Placebo: 35	Liraglutide 0.6 mg: 25.3 (3.6); Liraglutide 0.9 mg: 24.4 (3.4)	24.9 (4.0)	Liraglutide 0.3 mg QD (1 week) and 0.6 mg QD (23 weeks) or Liraglutide 0.3 mg QD (1 week), 0.6 mg QD (1 week) and 0.9 mg QD (22 weeks) + sulphonylureas	Placebo + sulphonylureas
GLP-1RA versus GLP-1RA study										
Ji *et al*. 2013^12^	340/338	China (Mainland and Taiwan), India, Japan, Korea	26	7.7 (5.1)/8.6 (6.0)	55 (11)/56 (10)	46.2/45.6	26.4 (3.7)	26.7 (3.4)	Exenatide 2 mg QW + OAD	Exenatide 5 ug BID (4 weeks) and 10 ug BID (22 weeks) + OAD

I, intervention; C, control; SD, standard deviation; BMI, body mass index; GLP-1RA, glucagon-like peptide-1 receptor agonist; QW, once a week; BID, twice a day; QD, once a day; OAD, oral anti-diabetic drug; CSII, continuous subcutaneous insulin infusion; NA, not applicable; TID, thrice a day.

### Quality of Bias Control

For the 20 studies in the meta-analysis, the results of a funnel plot (Fig. S1A) and Egger’s test (Fig. S1B) each revealed a potential publication bias (p = 0.002). The assessment results for each bias risk are described in Table S1.

### Comparisons of Glucose-lowering Efficacies and Weight-loss Effects

**Figure 2:**
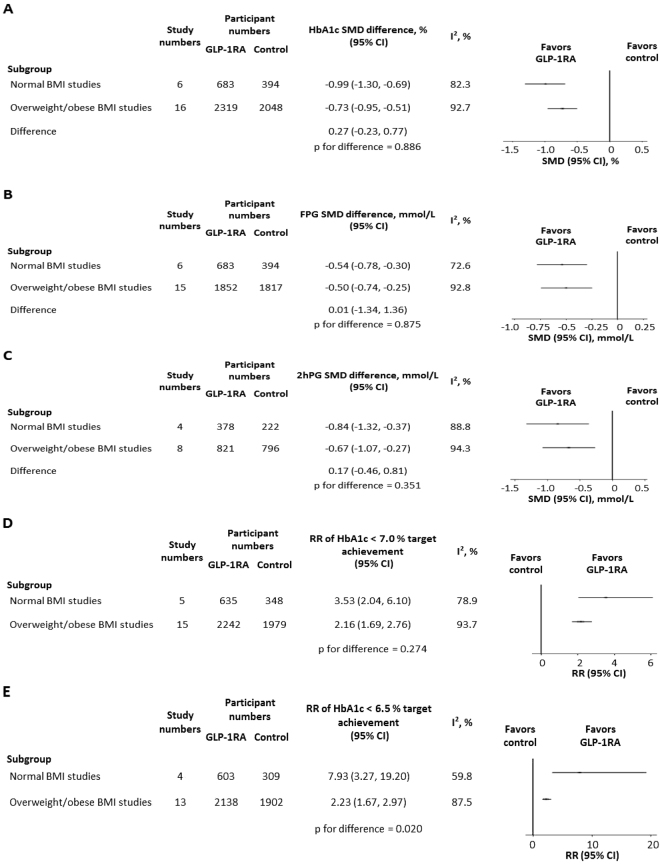
Comparisons between normal BMI Asian studies and overweight/obese BMI Asian studies in (**A**) HbA1c change, (**B**) fasting plasma glucose change, (**C**) 2-hour postprandial glucose change, (**D**) relative risk of HbA1c < 7.0% target achievement and (**E**) relative risk of HbA1c < 6.5% target achievement. BMI, body mass index; GLP-1RA, glucagon-like peptide-1 receptor agonist; SMD, standardized mean difference; CI, confidence interval; FPG, fasting plasma glucose; 2hPG, 2-hour postprandial glucose; RR, relative risk.

The overall SMD for HbA1c change was −0.81% (95% CI −0.99, −0.62; I^2^ = 91.5%). In the normal BMI Asian studies (n = 6), the HbA1c reduction from baseline was −0.99% (95% CI −1.30, −0.69; I^2^ = 82.3%) (Fig. 2A). In the overweight/obese BMI Asian studies (n = 16), HbA1c was reduced from baseline by −0.73% (95% CI −0.95, −0.51; I^2^ = 92.7%). The difference in HbA1c change was 0.27% between the two groups (95% CI −0.23, 0.77), but without statistical significance (p = 0.886). As there was only one study each of exenatide or lixisenatide in the normal BMI group^21,38^, we additionally analyzed the studies with liraglutide (n = 4 for normal BMI studies^20,22,23,39^ and n = 8 for overweight/obese BMI studies^28–30,32–34,37,39^). There was no significant difference in liraglutide-induced HbA1c reduction between the two groups (0.35%; 95% CI −0.23, 0.93; p = 0.770) (Fig. S2A). As the control patients received DPP-4 inhibitors, metformin, sulphonylureas, and insulin among others, we finally analyzed the studies that only compared GLP-1RA versus placebo^21,23,25,27,30–32,35,38,39^. Similar HbA1c changes were observed in the normal BMI studies (n = 4; −1.16%; 95% CI −1.32, −1.00; I^2^ = 58.0%)^21,23,38,39^ and the overweight/obese BMI studies (n = 8; −0.96%; 95% CI −1.05, −0.87; I^2^ = 91.1%)^25,27,30–32,35,38,39^ (p = 0.834) (Fig. S2B). We then divided our meta-analysis into normal BMI (n = 6)^20–23,38,39^, overweight BMI (n = 14)^24–27,29–35,37–39^, and obese BMI (n = 2)^28,36^ groups to further address the different HbA1c-lowering effects of GLP-1RAs. The results revealed no significant difference in GLP-1RA efficacies between the overweight and normal BMI groups (p = 0.395) or between the overweight and obese BMI groups (p = 0.255) (Fig. S2C). However, the HbA1c reduction in the normal BMI group tended to be superior to that in the obese BMI group, although the difference did not reach statistical significance (p = 0.056).

The overall SMD for FPG change from baseline was −0.51 mmol/L (95% CI −0.70, −0.33; I^2^ = 90.3%). No significant difference in FPG change was found between the normal BMI group (n = 6; −0.54 mmol/L; 95% CI −0.78, −0.30; I^2^ = 72.6%)^20–23,38,39^ and the overweight/obese BMI group (n = 15; −0.50 mmol/L; 95% CI −0.74, −0.25; I^2^ = 92.8%)^24–33,35–39^ (p = 0.875) (Fig. 2B).

A total of 11 trials described 2hPG outcomes, wherein three were assessed after a standard mixed meal^21,24,30^, seven were obtained from self-monitored blood glucose^20,23,28,29,31,35,39^, and one was examined by an oral glucose tolerance test^36^. The overall change in 2hPG was −0.74 mmol/L (95% CI −1.04, −0.44; I^2^ = 92.4%) across these trials. Similar 2hPG reductions were observed in the normal BMI studies (n = 4; −0.84 mmol/L; 95% CI −1.32, −0.37; I^2^ = 88.8%)^20,21,23,39^ and the overweight/obese BMI studies (n = 8; −0.67 mmol/L; 95% CI −1.07, −0.27; I^2^ = 94.3%)^24,28–31,35,36,39^ (p = 0.351) (Fig. 2C).

To calculate the RR of HbA1c < 7.0% target goal achievement for GLP-1RA versus control, five normal BMI trials^21–23,38,39^ and fifteen overweight/obese BMI trials^24–32,34–39^ were included. The overall RR for this HbA1c target goal achievement was 2.45 (95% CI 1.95, 3.10; I^2^ = 93.1%). The RR in the normal BMI studies (3.53; 95% CI 2.04, 6.10; I^2^ = 78.9%) tended to be equivalent to that in the overweight/obese BMI studies (2.16; 95% CI 1.69, 2.76; I^2^ = 93.7%) (p = 0.274) (Fig. 2D). In the trials with GLP-1RAs versus placebo, similar results were obtained in the normal BMI studies (n = 4; 4.08; 95% CI 2.49, 6.69; I^2^ = 55.9%)^21,23,38,39^ and the overweight/obese BMI studies (n = 8; 5.23; 95% CI 2.91, 9.43; I^2^ = 90.5%)^25,27,30–32,35,38,39^ (p = 0.648) (Fig. S2D).

The overall RR of HbA1c < 6.5% target goal achievement for GLP-1RA versus control was 2.82 (95% CI 2.09, 3.82; I^2^ = 87.9%). The RR was higher in the normal BMI group (n = 4; 7.93; 95% CI 3.27, 19.20; I^2^ = 59.8%)^21–23,39^ than that in the overweight/obese BMI group (n = 13; 2.23; 95% CI 1.67, 2.97; I^2^ = 87.5%)^24–27,29–32,34–37,39^ (p = 0.020) (Fig. 2E). Compared with the RR of GLP-1RA versus placebo in the overweight/obese BMI studies (n = 7; 4.30; 95% CI 2.46, 7.53; I^2^ = 73.7%)^25,27,30–32,35,39^, the RR in the normal BMI studies (n = 3; 11.34; 95% CI 5.73, 22.46; I^2^ = 0.0%)^21,23,39^ tended to be greater, but fell short of statistical significance (p = 0.051) (Fig. S2E).

The overall SMD for weight loss from baseline was −0.31 kg (95% CI −0.47, −0.15; I^2^ = 86.5%). There was no significant difference in body weight change between the normal BMI studies (n = 6; −0.03 kg; 95% CI −0.38, 0.32; I^2^ = 87.6%)^20–23,38,39^ and the overweight/obese BMI studies (n = 14; −0.44 kg; 95% CI −0.62, −0.26; I^2^ = 85.3%)^24–29,31–33,35–39^ (p = 0.572) (Fig. S2F).

### Sensitivity Analysis and Meta-regression Analyses

Intertrial heterogeneity was detected across the studies for HbA1c change. A sensitivity analysis was performed, but no obviously low-quality studies were found (Fig. S3). Furthermore, a trim-and-fill procedure did reveal any data changes and produced consistent results (p = 0.238). These findings indicated that the results for HbA1c change were robust and reliable.

Univariate meta-regression analysis was adopted to further investigate the source of the heterogeneity. The results of the meta-regression analysis are presented in Table 2. Longer duration of diabetes (p = 0.003) and lower percentage of females (p = 0.003) were found to be associated with greater reduction of HbA1c from baseline. Varied sample size (p = 0.026), optimistic conclusions (p = 0.002), and comparisons between GLP-1RA and placebo (p = 0.000) were significantly correlated with HbA1c change.

**Table 2:** Univariate meta-regression analysis results of HbA1c change.

	Estimate	95% CI	p value
Treatment duration	0.020	(−0.004, 0.043)	0.104
Diabetes duration	−0.083	(−0.136, −0.030)	0.003
Age	−0.012	(−0.047, 0.022)	0.466
Female percentage	0.041	(0.015, 0.066)	0.003
Baseline HbA1c level	0.070	(−0.493, 0.634)	0.800
Sample size	0.002	(0.000, 0.003)	0.026
Study with statistical significance	−0.855	(−1.364, −0.347)	0.002
Study with GLP−1RA versus placebo	−0.825	(−1.153, −0.496)	0.000

CI, confidence interval; GLP−1RA, glucagon-like peptide-1 receptor agonist.

### Safety Comparison

**Figure 3:**
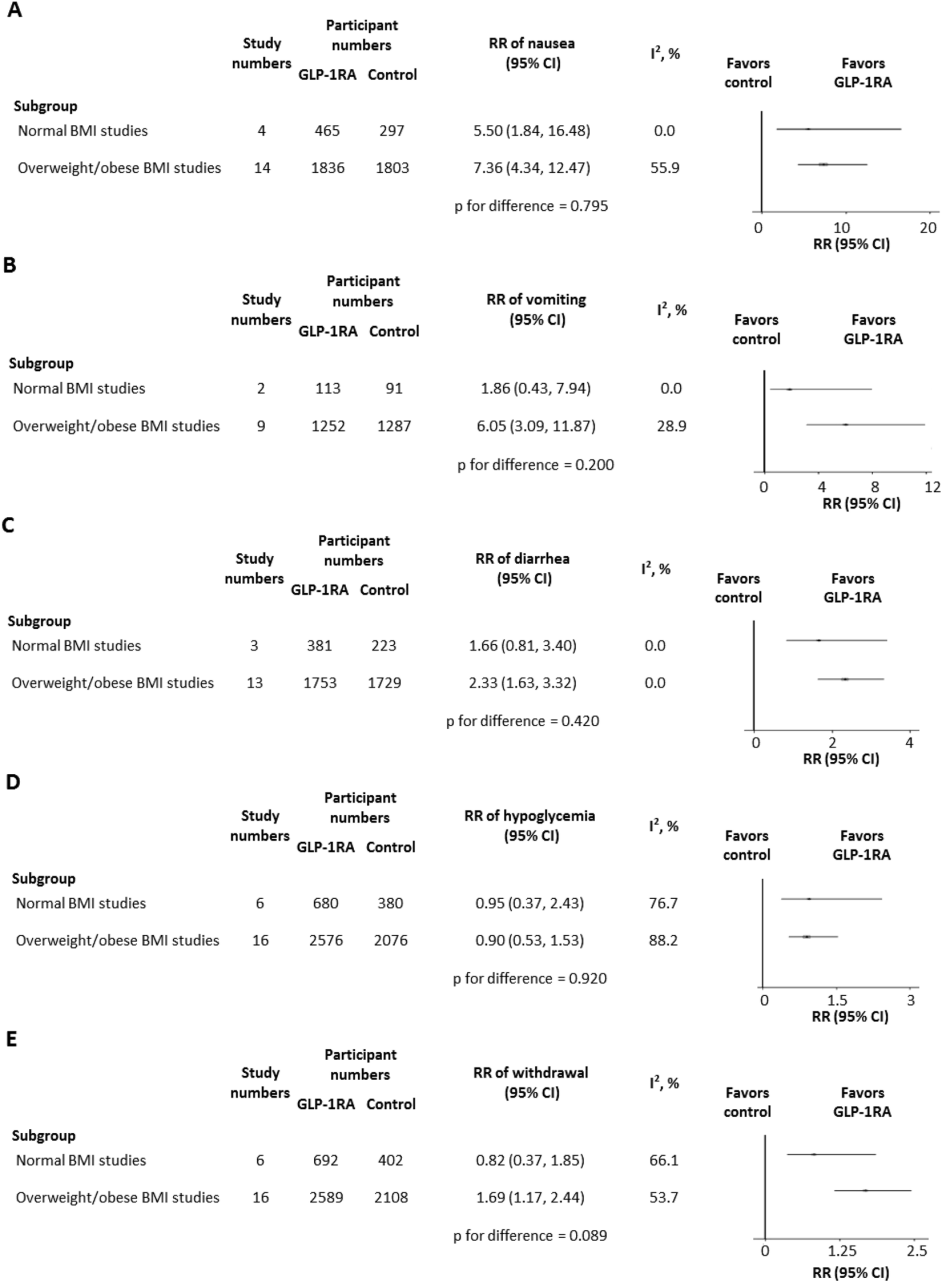
Comparisons between normal BMI Asian studies and overweight/obese BMI Asian studies in (**A**) nausea, (**B**) vomiting, (**C**) diarrhea, (**D**) hypoglycemia and (**E**) withdrawal. BMI, body mass index; GLP-1RA, glucagon-like peptide-1 receptor agonist; RR, relative risk; CI, confidence interval.

The RRs of adverse events in both the normal BMI studies and the overweight/obese BMI studies are depicted in Fig. 3. Compared with controlled anti-diabetic agents, GLP-1RA administration led to more gastrointestinal side effects, especially in the overweight/obese BMI trials. However, there was no significant difference in the RRs of nausea (p = 0.795) (Fig. 3A), vomiting (p = 0.200) (Fig. 3B), or diarrhea (p = 0.420) (Fig. 3C) between the two BMI groups. Intriguingly, similar hypoglycemic risks were observed for GLP-1RA and control agents, for which the RRs were also equivalent in the two BMI groups (p = 0.920) (Fig. 3D). The RR of withdrawal appeared to be higher in the overweight/obese BMI trials than that in the normal BMI trials, but without statistical significance (p = 0.089) (Fig. 3E). Owing to the limited data collection, the adverse events of constipation, pancreatitis, and cancer could not be compared.

## Discussion

From our meta-analysis, the HbA1c reductions from baseline were comparable among Asian T2D patients with and without overweight/obesity. While the rates of HbA1c < 7.0% target goal achievement were fair in the two BMI groups, the rate of HbA1c < 6.5% target goal achievement in the normal BMI studies was superior to that in the overweight/obese BMI studies. In general, based on the accompanying FPG and 2hPG reductions in the two groups, it is suggested that the glucose-lowering efficacies of GLP-1RAs are equivalent in Asian T2D patients, regardless of overweight/obesity.

A previous meta-analysis reported that HbA1c reduction by GLP-1 analogues was higher in studies with lower baseline BMI than that in studies with higher baseline BMI^5^. However, the cut-off point for BMI was set at 30 kg/m^2^, meaning that most patients in the lower baseline BMI studies were classified as overweight^25,27,38^. A Chinese RCT including 37 normal BMI, 37 overweight, and 26 obese T2D patients found that the hypoglycemic efficacy of liraglutide was positively correlated with BMI, being most effective in patients with obesity^40^. A retrospective cohort study involving four BMI categories, 25.0–29.9, 30.0–34.9, 35.0–39.9, and ≥40.0 kg/m^2^, observed that the HbA1c decrease by liraglutide did not vary significantly according to BMI classification in the United States^41^. In order to further address the different glucose-lowering effects of GLP-1RAs, we divided the studies into normal BMI, overweight BMI, and obese BMI groups in our meta-analysis. Although the hypoglycemic effects were similar among the three BMI groups, it was intriguing to find that the HbA1c reduction in the normal BMI patients tended to be greater than that in the obese patients. This finding was consistent with the previous meta-analysis^5^. Owing to the limited number of normal BMI trials and inconsistent conclusions, further studies involving both normal BMI and overweight/obese patients are needed, and could clarify whether BMI variation gives rise to different hypoglycemic efficacies of GLP-1RAs within patients of the same ethnicity.

GLP-1 contributes to weight loss by both decreasing food intake and increasing energy expenditure. It is reported that in lean subjects, supraphysiological infusions of GLP-1 increase energy expenditure but restrained by physiological insulin level^42^. In obese subjects, supraphysiological dose of GLP-1 reduces appetite and food intake, which subsequently induces weight loss, alleviates GLP-1 resistance, and increases insulin sensitivity^43^. As increased BMI impairs GLP-1 secretion and aggravates insulin resistance, the supraphysiological infusions of GLP-1 may act as a positive feedback signal in obese patients to improve insulin sensitivity and reduce chronic obesity. Although the underlying mechanism is only partly understood, several previous studies on liraglutide intervention reported that patients with higher BMI achieved greater body weight loss, suggesting that GLP-1RAs guarantee stable weight maintenance on lean patients^41,44,45^.

In this meta-analysis, compared with trial comparators, GLP-1 analogues had a significant weight advantage in the overweight/obese BMI studies. But in the normal BMI studies, the weight loss effects of GLP-1RAswere comparable to those of control agents. However, the difference in weight loss effect of GLP-1RAs between the two BMI groups was insignificant. It was assumed that the majority of comparator hypoglycemic drugs in the normal BMI studies were insulin and sulfonylureas, while the comparator agents in the overweight/obese BMI studies involved metformin, acarbose, and DPP-4 inhibitors, which have significant weight-loss effects. Therefore, the weight reduction efficacy of GLP-1RAs in the overweight/obese BMI studies may be narrowed down by these weight-losing comparator agents. Further studies are needed to explore how GLP-1RAs lead to different weight-loss effects between normal BMI and overweight/obese subjects.

Based on the meta-regression analysis, T2D duration and percentage of males were positively correlated with the hypoglycemic action of GLP-1RAs. These two factors were considered potential predictors of good response to GLP-1 analogues in another meta-analysis^5^. As the underlying mechanisms remain unclear, further studies are needed to investigate whether and how T2D duration and sex affect the efficacy of GLP-1RAs.

Regarding safety issues, gastrointestinal side effects, including nausea, vomiting, and diarrhea, seemed to be the most frequent adverse events in the GLP-1RAs treatment group. However, these events did not differ between the normal BMI and overweight/obese BMI studies. Compared with active reagents and placebo, GLP-1 analogues had a slight advantage for hypoglycemia occurrence. Similarly, the hypoglycemia risks with GLP-1RAs were similar among Asian T2D patients.

On account of their expensive cost and significant weight-loss effects, GLP-1RAs are more inclined to be rational medications for diabetes patients with overweight/obesity. In our meta-analysis, it was found that the glucose-lowering effects and safety of GLP-1RAs were equivalent in Asian T2D patients with and without overweight/obesity. The HbA1c reduction in the normal BMI patients tended to be superior to that in the obese patients. Moreover, GLP-1RAs usage in lean patients is beneficial for stable weight maintenance. Therefore, it is suggested that GLP-1RAs are optimal anti-diabetic medicines for T2D patients with normal BMI, being successful in both reducing glucose levels and maintaining a stable body weight.

A *post hoc* analysis examined the efficacy of liraglutide in Latino/Hispanic T2D individuals based on four phase Ш trials^46^. It was shown that Latino/Hispanic patients treated with liraglutide achieved significant HbA1c reductions versus the comparators of glimepiride, placebo, and sitagliptin respectively. In addition, 1.8 mg liraglutide caused a significant reduction in weight compared with glimepiride or sitagliptin. The baseline BMIs of subjects in the four trials were categorized into overweight and obesity. However, it did not investigate whether the differences in HbA1c and weight reductions were attributable to BMI variation. Further studies are needed to understand the effect of GLP-1RAs in other ethnic populations.

Based on the published patient profiles, our work is the first systematic review of RCTs to assess whether GLP-1RAs have different glucose-lowering efficacies and weight-loss effects in Asian T2D patients with varied BMI categories. The majority of the included studies had adequate randomization and accounted for the intention-to-treat population in their results. Moreover, all of the included trials had sufficient exposure durations to retrieve glucose changes of clinical significance. However, several limitations to this meta-analysis need to be addressed. First, the racial heritages of the individuals in normal BMI studies were all Japanese. We used the same search term and strategy to identify relevant trials published in Chinese. However, all of the Chinese reports were found to only involve overweight/obese T2D patients (data not shown). With the combination of screened trials described in English, it was revealed that GLP-1RAs were much more prone to be applied for treatment of overweight/obese T2D patients than for non-obese T2D patients. Second, among the several GLP-1RAs in clinical use, only exenatide, liraglutide and lixisenatide were included in our meta-analysis. This was because other GLP-1 analogues, such as dulaglutide and loxenatide, were only used in overweight/obese BMI trials. Third, the studies included in the meta-analysis displayed a potential publication bias. We searched the literatures in various databases to collect as many relevant trials as possible. Although we narrowed down our inclusion criteria to only accept reports published in English, which may have improved quality, studies with optimistic results were still more favored for publication than those with negative findings. Fourth, intertrial heterogeneity existed across the studies on HbA1c change. A sensitivity analysis and trim-and-fill procedure was performed to verify that the results were robust and reliable. In addition, it was shown that the factors of diabetes duration, sex percentage, study sample size, studies with significant results, and comparisons between GLP-1RAs and placebo could clarify the sources of the heterogeneity.

Owing to the weight-loss effect, GLP-1RA treatment is preferred for diabetes patients with overweight/obesity. Our analysis revealed that in Asia, the glucose-lowering efficacies of GLP-1RAs were equivalent in T2D patients with and without overweight/obesity. The HbA1c < 6.5% target goal achievement in the normal BMI patients was superior to that in the overweight/obese patients. Moreover, on account of their stable weight maintenance and hypoglycemia risk advantage, GLP-1 analogues were considered an optimal choice for Asian T2D patients with normal BMI. To investigate whether varied BMI categories can affect the hypoglycemic and weight-loss effects of GLP-1RAs, further studies conducted in different ethnicities are warranted. Overall, our findings can contribute to guidance for rational GLP-1RA usage in Asia,suggesting that GLP-1 analogues are desirable for T2D patients with normal BMI as well as for those with overweight/obesity.

## Electronic supplementary material

Supplementary Information
