# Supplementary material for: Glucagon-like peptide-1 mimetics, optimal for Asian type 2 diabetes patients with and without overweight/obesity: meta-analysis of randomized controlled trials
Source: Sci Rep. 2017 Nov 22;7:15997. doi: 10.1038/s41598-017-16018-9 (PMC5700049; doi:10.1038/s41598-017-16018-9)
Supplement: Supplementary file 1 — Supplementary Information [file 41598_2017_16018_MOESM1_ESM.pdf]

Glucagon-like peptide-1 mimetics, optimal for Asian type 2 diabetes patients with and without overweight/obesity: meta-analysis of randomized controlled trials

Fang Zhang, Lizhi Tang, Yuwei Zhang, Qingguo Lü, Nanwei Tong*

*Corresponding author

Table S1. Risk of bias assessment for the included studies

study	Random sequence generation	Allocation concealment	Blinding of participants and personnel	Blinding outcome assessment	Incomplete outcome data	Selective reporting	Other bias
Araki et al. 2015 [17]	low risk	low risk	high risk	high risk	low risk	low risk	low risk
Xu et al. 2014 [24]	low risk	unclear	high risk	high risk	low risk	low risk	low risk
Chen et al. 2017 [18]	low risk	low risk	low risk	low risk	low risk	low risk	low risk
Gao et al. 2009 [25]	low risk	unclear	low risk	low risk	low risk	low risk	low risk
Inagaki et al. 2012 [26]	low risk	low risk	high risk	high risk	low risk	low risk	low risk
Kadowaki et al. 2011 [27]	low risk	low risk	low risk	low risk	low risk	low risk	low risk
Kadowaki et al. 2009 [38]	low risk	unclear	low risk	low risk	low risk	low risk	low risk
Kaku et al. 2010 [39]	low risk	unclear	low risk	low risk	low risk	low risk	low risk
Ke et al. 2015 [13]	low risk	unclear	high risk	high risk	high risk	low risk	low risk
Li et al. 2012 [28]	low risk	unclear	high risk	high risk	high risk	low risk	low risk
Li et al. 2014 [29]	low risk	unclear	low risk	low risk	low risk	low risk	low risk
Miyagawa et al. 2015 [30]	low risk	low risk	low risk	low risk	low risk	low risk	low risk
Seino et al. 2012 [31]	low risk	unclear	low risk	low risk	low risk	low risk	low risk
Seino et al. 2016 [32]	low risk	unclear	low risk	low risk	low risk	low risk	low risk
Shi et al. 2017 [14]	low risk	unclear	high risk	high risk	high risk	low risk	low risk
Takeshita et al. 2015 [33]	low risk	low risk	high risk	high risk	high risk	low risk	low risk
Tanaka et al. 2015 [15]	low risk	unclear	high risk	high risk	high risk	low risk	low risk
Terauchi et al. 2014 [19]	low risk	unclear	low risk	low risk	low risk	low risk	low risk
Yang et al. 2011 [34]	low risk	unclear	low risk	low risk	high risk	low risk	low risk
Yoon et al. 2017 [16]	low risk	unclear	high risk	high risk	low risk	low risk	low risk

Table S1. Continued

Study	Random sequence generation	Allocation concealment	Blinding of participants and personnel	Blinding outcome assessment	Incomplete outcome data	Selective reporting	Other bias
Pan et al. 2014 [35]	low risk	unclear	low risk	low risk	low risk	low risk	low risk
Yuan et al. 2012 [36]	low risk	unclear	high risk	high risk	low risk	low risk	low risk
Zang et al. 2016 [37]	low risk	low risk	high risk	high risk	low risk	low risk	low risk
Inoue et al. 2015 [20]	unclear	unclear	high risk	high risk	low risk	low risk	low risk
Onishi et al. 2015 [21]	unclear	low risk	low risk	low risk	low risk	low risk	low risk
Seino et al. 2010 [22]	low risk	unclear	low risk	low risk	low risk	low risk	low risk
Seino et al. 2008 [23]	low risk	low risk	low risk	low risk	low risk	low risk	low risk
Ji et al. 2013 [12]	low risk	unclear	high risk	high risk	low risk	low risk	low risk

A

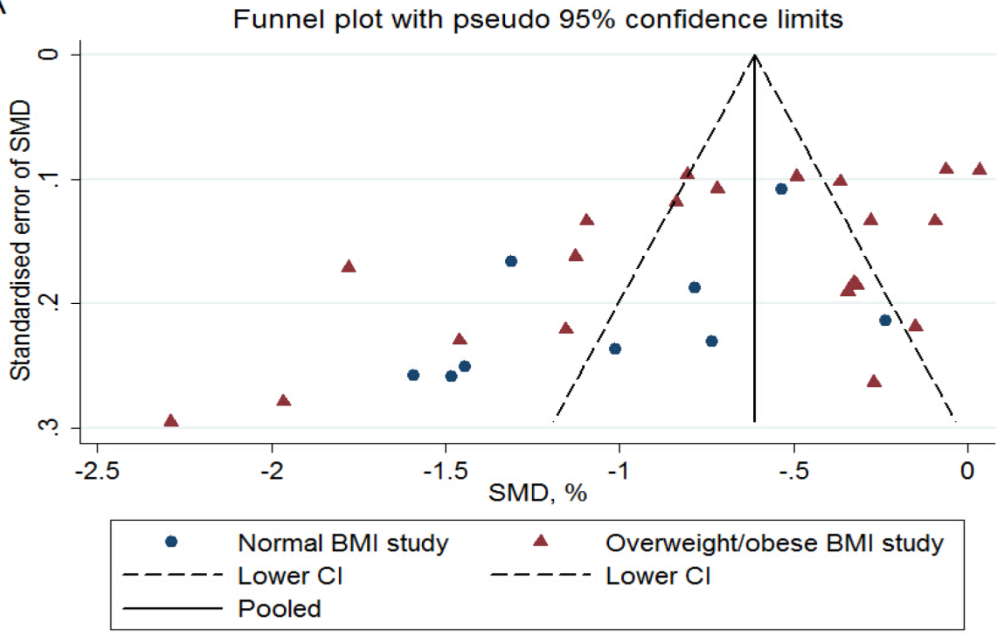

B

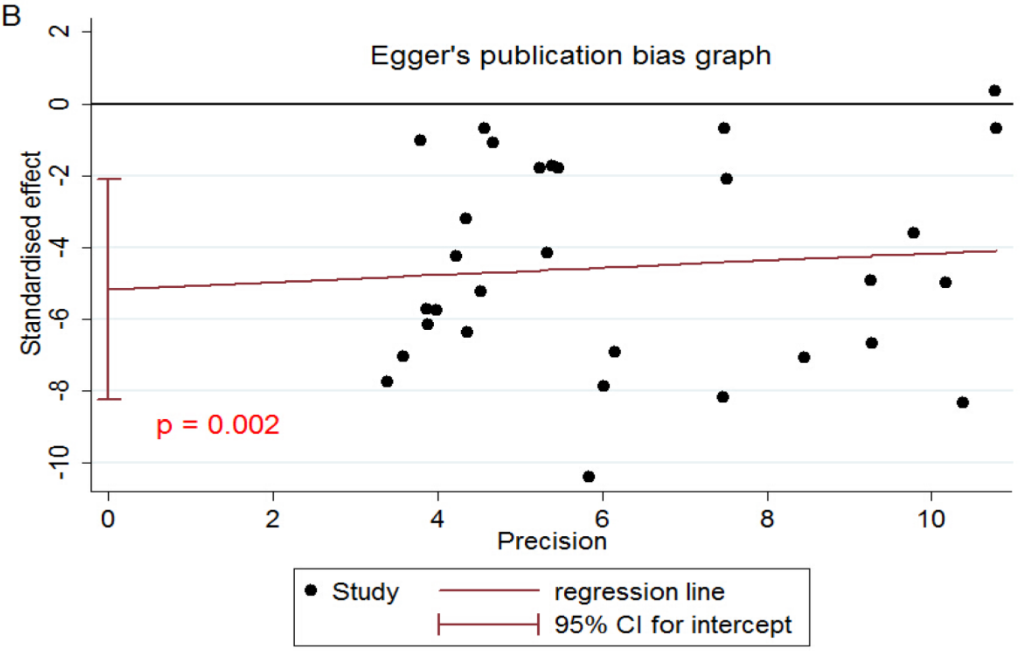

Fig. S1. Funnel plot and Egger's test graph of HbA1c change in meta-analysis. (A) Funnel plot; (B) Egger's test graph. *Studies with subgroups of diverse-dose GLP-1RA treatments or varied controlled agents were divided into respective comparison pairs: each pair included only one dose GLP-1RA versus one controlled comparator.

A

Subgroup	Study numbers	Participant numbers Liraglutide	Control	HbA1c SMD difference, % (95% CI)	I^2, %
Normal BMI studies	4	570	303	-0.96 (-1.33, -0.59)	84.6
Overweight/obese BMI studies	9	1159	920	-0.62 (-0.97, -0.26)	94.1
Difference				0.35 (-0.23, 0.93)	
				p for difference = 0.770	

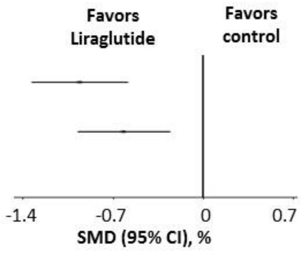

B

Subgroup	Study numbers	Participant numbers GLP-1RA	Placebo	HbA1c SMD difference, % (95% CI)	I^2, %
Normal BMI studies	4	372	217	-1.16 (-1.32, -1.00)	58.0
Overweight/obese BMI studies	8	1154	946	-0.96 (-1.05, -0.87)	91.1
Difference				0.05 (-0.58, 0.48)	
				p for difference = 0.834	

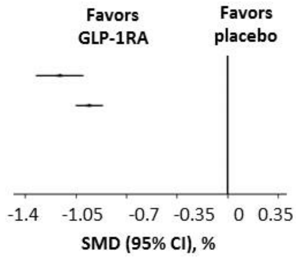

C

Subgroup	Study numbers	Participant numbers GLP-1RA	Control	HbA1c SMD difference, % (95% CI)	I^2, %
Normal BMI studies	6	683	394	-0.99 (-1.30, -0.69)	82.3
Overweight BMI studies	14	2244	1980	-0.78 (-1.02, -0.54)	93.3
Obese BMI studies	2	75	68	-0.20 (-0.53, 0.13)	0.0
p for difference among three groups = 0.130					

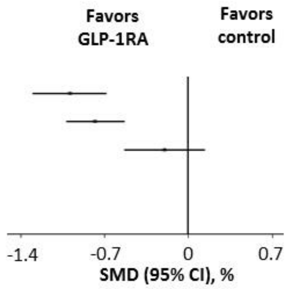

D

Subgroup	Study numbers	Participant numbers GLP-1RA	Placebo	RR of HbA1c < 7.0 % target achievement (95% CI)	I^2, %
Normal BMI studies	4	367	216	4.08 (2.49, 6.69)	55.9
Overweight/obese BMI studies	8	1135	935	5.23 (2.91, 9.43)	90.5
				p for difference = 0.648	

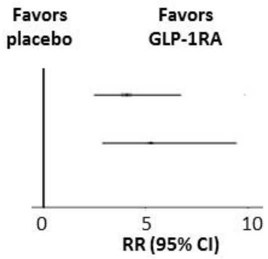

E

Subgroup	Study numbers	Participant numbers GLP-1RA	Placebo	RR of HbA1c < 6.5 % target achievement (95% CI)	I^2, %
Normal BMI studies	3	335	177	11.34 (5.73, 22.46)	0.0
Overweight/obese BMI studies	7	1070	900	4.30 (2.46, 7.53)	73.7
				p for difference = 0.051	

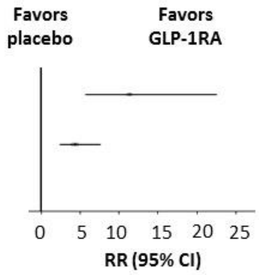

F

Subgroup	Study numbers	Participant numbers GLP-1RA	Participant numbers Control	Body weight SMD difference, kg (95% CI)	I^2, %
Normal BMI studies	6	680	392	-0.03 (-0.38, 0.32)	87.6
Overweight/obese BMI studies	14	1715	1747	-0.44 (-0.62, -0.26)	85.3
Difference				-0.40 (-0.76, 0.05)<br>p for difference = 0.572	

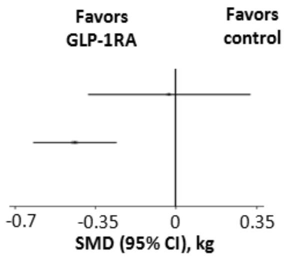

Fig. S2. Comparisons between normal BMI Asian studies and overweight/obese BMI Asian studies in (A) HbA1c change in liraglutide research, (B) HbA1c change in placebo-controlled studies, (C) HbA1c change in three BMI groups, (D) relative risk of HbA1c < 7.0% target achievement in placebo-controlled research, (E) relative risk of HbA1c < 6.5% target achievement in placebo-controlled research and (F) body weight change. BMI, body mass index; GLP-1RA, glucagon-like peptide-1 receptor agonist; SMD, standardized mean difference; CI, confidence interval; RR, relative risk.

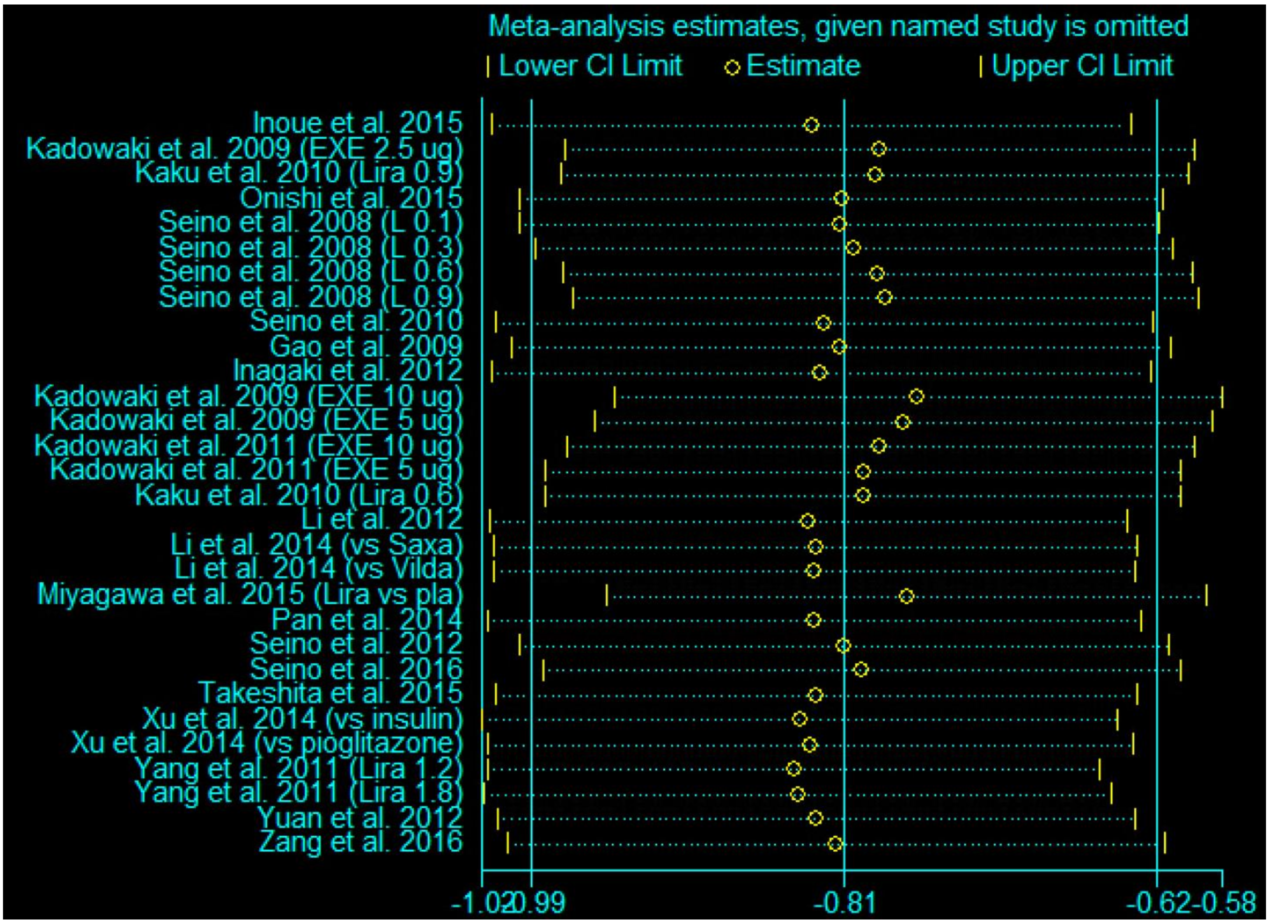

Fig. S3. Sensitivity analysis of studies included HbA1c change in meta-analysis.
